# Troponin T in stable patients with penetrating chest trauma: A diagnostic study

**DOI:** 10.4102/jcmsa.v4i1.402

**Published:** 2026-07-08

**Authors:** Nafi Y. Alam, Faatimah A. Asmal, Mikaela de Ponte, Yusuf E. Patel, Malaaika I. Siddique, Bongumusa Sithole, Dineo Mphago, Samukelisiwe Mpembe, Maeyane S. Moeng, Estelle Laney, Shumani Makhadi

**Affiliations:** 1 School of Clinical Medicine, Faculty of Health Sciences, University of the Witwatersrand, Johannesburg, South Africa

**Keywords:** troponin T, penetrating chest trauma, cardiac injury, occult injury, diagnostic accuracy

## Abstract

**Background:**

Penetrating chest trauma is a leading cause of mortality in South Africa. In haemodynamically stable patients with injuries within the cardiac box, occult cardiac injury remains difficult to detect, particularly in resource-limited settings. The diagnostic utility of troponin T in this population is poorly defined.

**Methods:**

A retrospective cohort study was conducted on haemodynamically stable patients presenting with penetrating anterior chest injuries to Charlotte Maxeke Johannesburg Academic Hospital between May 2022 and June 2024. Sensitivity, specificity, positive predictive value, and negative predictive value (NPV) of serum troponin T were calculated against subxiphoid pericardial window, surgical findings, ultrasound and electrocardiography.

**Results:**

Among 256 patients (96% male; median age 31 years), 65% sustained stab wounds and 35% gunshot wounds. Serum Troponin T demonstrated high sensitivity (90.5% pooled against definitive modalities) and an NPV of up to 88.8% in concordance analysis with commonly used screening modalities (non-reference comparators); NPV against definitive reference standards ranged from 50.0% to 66.7%, but had low specificity (37.5% pooled). Overall mortality was 3.9% and was significantly associated with haemopericardium (*p* = 0.020) and pleural injury (*p* = 0.029).

**Conclusion:**

Serum Troponin T offered high sensitivity and NPV for excluding occult cardiac injury in haemodynamically stable patients, supporting its role as an accessible screening adjunct in resource-constrained emergency departments.

**Contribution:**

This study represents the largest cohort evaluating serum troponin T in haemodynamically stable penetrating chest trauma, addressing a critical evidence gap. The findings are directly relevant to South African clinical practice and align with the journal’s focus on improving patient care in resource-limited settings.

## Introduction

Gunshot injuries carry significantly higher mortality compared to stab wounds.^1,2,3,4,5,6,7^

The cardiac box, anatomically bordered by the clavicles superiorly, the costal margins inferiorly and the midclavicular lines laterally, defines the region in which Penetrating Traumatic Cardiac Injury (PTCI) carry the highest risk of cardiac involvement.^8^ Injuries range from small myocardial contusions that may resolve with resuscitation,^4^ through coronary artery transection with haemopericardium and circulatory collapse,^9^ to full-thickness chamber damage causing acute cardiac tamponade.^10^ The right ventricle, being the most anterior chamber, is most affected.^5,9^ Cardiac tamponade and hypovolaemic shock secondary to haemorrhage remain the principal causes of PTCI-related fatality.^11^

In haemodynamically stable patients with a high suspicion of occult cardiac injury, diagnosis is challenging because clinical deterioration can occur rapidly.^12^ Available screening tools include erect chest radiography, extended focused assessment with sonography for trauma (eFAST), electrocardiography (ECG), echocardiography and cardiac biomarkers such as troponin T.^5^ The subxiphoid pericardial window remains the gold standard for identifying penetrating cardiac injuries^5,12^; however, its use requires theatre access and specialist expertise, rendering it impractical in many settings. Ultrasound is limited by operator proficiency, pneumopericardium, left haemothorax and patient habitus,^12,13^ and the J-wave on ECG, although associated with cardiac injury, has limited sensitivity.^13,14^ Meanwhile, arterial blood gas markers such as lactate and base deficit have demonstrated utility primarily in unstable populations; lactate alone or combined with the shock index can identify patients requiring surgical intervention, whereas base deficit offers little additional value.^15,16^

High-sensitivity troponin T is defined by two criteria: (1) a total coefficient of variance at the 99th percentile of ≤ 10%, and (2) measurability in at least 50% of healthy individuals at concentrations below the 99th percentile. These defining characteristics confer clinical advantages, including early detection of myocardial injury and high analytical precision at low concentrations, which enables reliable detection of small concentration changes over short intervals. This precision supports the application of rapid diagnostic algorithms such as the 0h/1h and 0h/3h protocols used in the evaluation of suspected acute coronary syndromes.

The test requires only a serum or plasma sample (depending on the laboratory). In this context, troponin T represents a potentially valuable diagnostic adjunct. The Elecsys^®^ Troponin T high-sensitivity (hs-cTnT) laboratory assay (Roche Diagnostics) is available in South African tertiary centres through the National Health Laboratory Service, and the cost is relatively low at R168.81 (approximately 9.47 USD).^17,18^ Although troponins are established as the biochemical gold standard for diagnosing acute coronary syndromes and have a recognised role in blunt cardiac trauma,^19,20,21^ their utility in penetrating chest trauma remains poorly defined. Case reports have demonstrated that troponin elevations may detect occult cardiac injury even when echocardiography is non-diagnostic,^8,19^ and a recent single-centre South African study reported high specificity (98%) but low sensitivity (19.7%) for troponins in this setting.^5^ Given the small sample sizes and conflicting results in the existing literature, a significant knowledge gap persists regarding the diagnostic accuracy of troponin T in haemodynamically stable patients with PTCI.

The aim of this study was to evaluate the diagnostic accuracy of troponin T for detecting cardiac injury in haemodynamically stable patients with penetrating chest trauma presenting to Charlotte Maxeke Johannesburg Academic Hospital (CMJAH). The specific objectives were: (1) to determine the sensitivity, specificity, positive predictive value (PPV) and negative predictive value (NPV) of troponin T compared with subxiphoid pericardial window, surgical findings, ultrasound and ECG; (2) to describe the demographics, injury mechanisms and anatomical distribution of injuries; and (3) to evaluate patient outcomes including length of hospital stay, complications and mortality.

## Research methods and design

### Study design and setting

A retrospective cohort study was undertaken. The research was carried out at the trauma unit of CMJAH, a Level 1 trauma centre affiliated with the University of the Witwatersrand, Johannesburg, South Africa. Charlotte Maxeke Johannesburg Academic Hospital functions as a primary referral centre for Gauteng province and handles many penetrating trauma cases, including those referred from nearby district hospitals and primary healthcare clinics.

### Study population and sampling strategy

The study population comprised all patients aged 18 years and older, of all sexes, presenting with confirmed penetrating anterior chest injuries within the cardiac box who were haemodynamically stable (systolic blood pressure > 90 mmHg) over a 2-year period from 01 May 2022 to 30 June 2024. Consecutive sampling was employed. Eligible patients were required to have baseline serum troponin T (Elecsys^®^ hs-cTnT, Roche Diagnostics; positive ≥14 ng/L, negative < 14 ng/L [99th percentile upper reference limit]) measured on presentation in the emergency department, and to have accessible, complete medical records. Only patients managed according to the hospital’s standardised guidelines for penetrating chest injuries, with recorded outcomes including length of hospitalisation, surgical interventions, complications and mortality, were included. Patients who were haemodynamically unstable (systolic blood pressure < 90 mmHg) requiring immediate surgery, pregnant patients and those with bottle-related stab wounds were excluded.

### Data collection

Data were collected retrospectively using a structured data collection sheet. Information was extracted from CMJAH’s hospital databases, including Medibank, REDCap and patient clinical files. Variables included demographics, mechanism and anatomical location of injury, vital signs on presentation, arterial blood gas parameters (lactate, base excess), serum troponin T results (baseline value on emergency department presentation and serial measurements where available; serial testing was not protocolised and was performed at the treating clinician’s discretion), imaging findings (chest radiography, eFAST), ECG results, subxiphoid pericardial window findings, intraoperative cardiac findings and clinical outcomes (length of hospital stay, complications, mortality). Troponin T was measured using the Elecsys^®^ Troponin T high-sensitivity laboratory assay (Roche Diagnostics, Germany) on blood samples collected in the emergency department on presentation. Levels below 14 ng/L were defined as negative and levels ≥ 14 ng/L as positive (99th percentile upper reference limit per manufacturer).

### Data analysis

Categorical variables were expressed as frequencies and percentages. Continuous variables were expressed as means with standard deviations (s.d.) for normally distributed data and medians with interquartile ranges (IQR) for non-normally distributed data; normality was assessed using the Shapiro–Wilk test. Associations between categorical variables were tested using the chi-square test or Fisher’s exact test as appropriate. The Mann–Whitney U test was applied for comparisons of continuous variables between groups. Cox proportional hazards regression was used to assess associations between sociodemographic and clinical factors and mortality, with unadjusted hazard ratios (HR) and 95% confidence intervals (CI) reported. Length of hospital stay was analysed as a count outcome; given evidence of overdispersion, negative binomial regression was applied to obtain incidence rate ratios (IRR) with 95% CI. Diagnostic accuracy of troponin T was evaluated by calculating sensitivity, specificity, PPV and NPV against each reference standard separately: (1) subxiphoid pericardial window findings (positive = blood in the pericardial sac at the time of the procedure); (2) intraoperative cardiac findings at thoracotomy or laparotomy (positive = confirmed myocardial or coronary artery injury); (3) eFAST (positive = pericardial effusion on bedside ultrasound); and (4) ECG (positive = ST-segment changes, T-wave inversion, or J waves). Patients who underwent more than one reference test appear in each respective 2 × 2 table; no composite reference standard was constructed, and results are reported per comparator to avoid double-counting. All analyses were performed using Stata version 18 (StataCorp, College Station, Texas, United States). A *p*-value of less than 0.05 was considered statistically significant.

### Ethical considerations

Ethical clearance to conduct this study was obtained from the University of the Witwatersrand Research Ethics Committee (No. M2410105). Institutional approval was granted by the Chief Executive Officer of CMJAH. The study was conducted in accordance with the principles of the Declaration of Helsinki. As this was a retrospective review of de-identified hospital records, individual patient consent was waived by the ethics committee.

## Results

**FIGURE 1:**
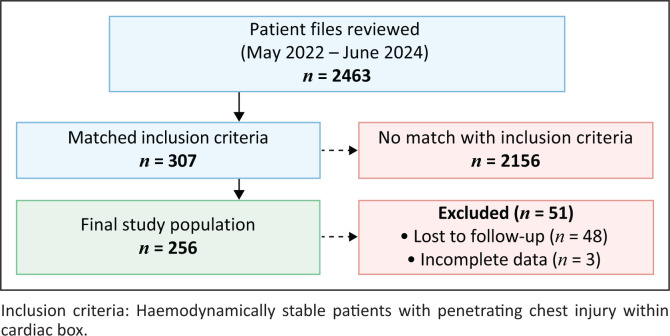
Patient recruitment flow diagram.

A total of 256 patients with penetrating chest trauma met the inclusion criteria (Figure 1). The majority were male (*n* = 246/256, 96.1%), with a median age of 31 years (IQR 26–37) (Table 1). Injuries occurred more frequently on weekends (Saturday: *n* = 57/256, 22.3%; Sunday: *n* = 66/256, 25.9%), and 76.2% (*n* = 195/256) occurred after weekday working hours. Most patients (*n* = 148/256, 57.8%) were referred to CMJAH, predominantly from clinics (*n* = 89/148, 60.1%) and other hospitals (*n* = 59/148, 39.9%). The majority of admitted patients (*n* = 159/256, 62.1%) had a median hospital stay of 3 days (IQR 1–6). Physiologically, most patients were stable, with a median systolic blood pressure of 124 mmHg (IQR 113–135.5), although metabolic derangements were noted (median lactate 2.7 mmol/L, IQR 1.7–4.15; median base excess −4.1 mmol/L, IQR −6.5 to −1.9) (Table 1).

**TABLE 1:** Demographics and physiological parameters.

Variable	Median	IQR
Age (years)	31	26–37
**Vitals**		
Systolic BP (mmHg)	124	113–135.5
Diastolic BP (mmHg)	79	70–86
Lactate (mmol/L)	2.7	1.7–4.15
Base excess (mmol/L)	−4.1	−6.5 – -1.9
Chest Abbreviated Injury Scale (AIS)	32	-

IQR, interquartile range; BP, blood pressure.

Stab wounds accounted for 65.2% (*n* = 167/256) of injuries, while gunshot wounds comprised 34.8% (*n* = 89/256) (Table 2). The left chest was the most common site of injury (*n* = 122/256, 47.7%), followed by the right chest (*n* = 85/256, 33.2%). Only 13.7% (*n* = 35/256) of patients had multiple or complex anatomical involvement, with sternal injuries present in 12.5% (*n* = 32/256).

**TABLE 2:** Mechanism and anatomical location of injury.

Injury	*n*	%
**Mechanism**		
Stab wound	167	65
Gunshot wound	89	35
**Anatomical location**		
Left chest	122	48
Right chest	85	33
Sternum	13	5
Left and right chest	17	6
Left chest and sternum	7	3
Right chest and sternum	12	5

Associated pleural injuries were highly prevalent (96%), including haemopneumothorax (44%), haemothorax (35%) and pneumothorax (17%) identified on chest radiography. Pericardial involvement was rare (4%), with cardiac tamponade documented in 1% and haemopericardium in 3% of patients.

Serial measurements were obtained in a subset of patients (*n* = 14/256, 5.5%) at the treating clinician’s discretion; among these, (*n* = 11) showed a rising troponin T concentration (defined as a delta change of ≥ 5 ng/L, consistent with European Society of Cardiology recommendations for acute coronary syndrome). Baseline troponin was higher among non-survivors (median 31 ng/L versus 11 ng/L in survivors), although this difference did not reach statistical significance (*p* = 0.181).

Serum Troponin T, therefore, identified most patients with true cardiac injury when evaluated against definitive reference standards (Table 3 and Table 4); however, specificity was low, and a negative result carries a moderate risk of false negativity relative to these definitive standards.

**TABLE 3:** Troponin T versus subxiphoid pericardial window findings.

Troponin T results	Subxiphoid positive	Subxiphoid negative	Row total
Troponin T positive	6	2	8
Troponin T negative	1	2	3

**Total**	**7**	**4**	**11**

Note: Calculated: Sensitivity 85.7% (6/7), Specificity 50.0% (2/4), PPV 75.0% (6/8), NPV 66.7% (2/3).

PPV, positive predictive value; NPV, negative predictive value.

**TABLE 4:** Troponin T versus cardiac surgery findings.

Troponin T results	Cardiac surgery positive	Cardiac surgery negative	Row total
Troponin T positive	13	3	16
Troponin T negative	1	1	2

**Total**	**14**	**4**	**18**

Note: Calculated: Sensitivity 92.9% (13/14), Specificity 25.0% (1/4), PPV 81.3% (13/16), NPV 50.0% (1/2). Some patients may appear in both Table 3 and Table 4 if they underwent both procedures.

PPV, positive predictive value; NPV, negative predictive value.

When troponin T results were compared with commonly used non-reference screening modalities (Table 5), concordance analysis showed moderate agreement with ultrasound (sensitivity 73.5%, NPV 88.8%) and lower agreement with ECG (sensitivity 46.2%, NPV 78.8%). These figures reflect inter-test agreement and should not be interpreted as true diagnostic accuracy measures; the formal diagnostic accuracy of troponin T against validated reference standards is presented in Table 3 and Table 4.

**TABLE 5:** Concordance of Troponin T with commonly used screening modalities (non-reference comparators).

Comparator	Sensitivity (%)	Specificity (%)	PPV (%)	NPV (%)
Ultrasound	73.5	44.1	21.7	88.8
ECG	46.2	45.6	16.2	78.8
Lactate	66.3	45.9	45.6	66.7
Base excess	60.1	41.9	75.8	25.7

Note: The comparators in this table (eFAST ultrasound, ECG, lactate, base excess) are not validated reference standards for penetrating cardiac injury. The metrics reported reflect inter-test concordance rather than true diagnostic accuracy per STARD 2015 guidelines. Formal diagnostic accuracy against reference standards (subxiphoid pericardial window and intraoperative findings) is reported in Table 3 and Table 4.

PPV, positive predictive value; NPV, negative predictive value; ECG, electrocardiography; STARD, standards for reporting diagnostic accuracy studies.

Among the 256 patients, ECG was performed in 32.0% (*n* = 82/256), of which 80.5% (*n* = 66/82) were normal. Extended focused assessment with sonography for trauma was performed in (*n* = 256/256, 100%) of patients. These utilisation rates reflect the cohort’s stable baseline and the after-hours presentation pattern, during which access to ancillary investigations was limited.

In a negative binomial regression model of 150 patients (Table 6), higher troponin and lactate levels showed borderline associations with increased hospital length of stay. Each 1 ng/L increase in troponin was associated with a 0.22% longer stay (IRR 1.0022, 95% CI 0.9998–1.0047; *p* = 0.073), while each 1 mmol/L increase in lactate was associated with a 12% longer stay (IRR 1.12, 95% CI 0.997–1.259; *p* = 0.057). Base excess was not significantly associated with length of stay (IRR 1.04, 95% CI 0.97–1.12; *p* = 0.311). The constant term corresponded to a baseline expected length of stay of 3.1 days.

**TABLE 6:** Negative binomial regression of predictors of hospital length of stay (*N* = 150).

Predictor	IRR	95% CI	*p*-value
Troponin (ng/L)	1.0022	0.9998–1.0047	0.073
Lactate (mmol/L)	1.1202	0.9967–1.2591	0.057
Base excess (mmol/L)	1.0385	0.9652–1.1174	0.311
Constant	3.0695	2.1589–4.3642	< 0.001

CI, confidence interval; IRR, incidence rate ratios.

Overall mortality was low (3.9%, *n* = 10/256), markedly lower than historic reports of penetrating cardiac injuries, reflecting the selection of haemodynamically stable patients. Deaths were significantly associated with haemopericardium (40% mortality; *p* = 0.020) and pleural injuries (*p* = 0.029), as well as abnormal lactate and base excess values. Cox regression analysis identified age (HR 1.12 per year, *p* = 0.03) and radiological evidence of haemothorax or haemopneumothorax (HR > 10^8^, *p* < 0.001) as strong independent predictors of mortality. The mechanism of injury (stab versus gunshot wound) was not independently significant.

## Discussion

In this retrospective cohort study of 256 haemodynamically stable patients with penetrating chest trauma, troponin T demonstrated high sensitivity (85.7% – 92.9% against definitive modalities; 90.5% pooled) for detecting occult cardiac injury when evaluated against validated reference standards (subxiphoid pericardial window and intraoperative findings) (Table 3 and Table 4). Negative predictive value against these definitive modalities ranged from 50.0% to 66.7%; concordance analysis with non-reference screening modalities (Table 5) showed NPV up to 88.8%, though these figures reflect inter-test agreement rather than true diagnostic performance. Specificity was low (25.0% – 50.0% against definitive modalities; 37.5% pooled), reflecting non-specific troponin elevations in the context of associated thoracic trauma. Overall mortality was 3.9% and was significantly associated with pericardial effusion and pleural injury, rather than with biochemical markers.

Over 90% of global deaths because of interpersonal violence occur in low- and middle-income countries (LMICs), where structural challenges such as limited pre-hospital care, inequitable emergency services and shortages of trained personnel contribute to the high trauma burden.^22,23^ Our cohort, comprising predominantly young males with injuries occurring after hours and on weekends, mirrors the well-documented South African trauma pattern.^5,24^ Stab wounds were the predominant mechanism, consistent with regional literature reflecting the greater availability and lower cost of knives in LMICs.^5,24^ Gunshot wounds, although more lethal, are associated with higher pre-hospital mortality and consequently fewer hospital presentations.^4,5^

As reported in the results, ECG was underutilised; ECG was performed in a minority of patients (32.0%) and was mostly normal when performed (80.5%), reinforcing its limited diagnostic yield in PTCI. The subxiphoid pericardial window, although the diagnostic gold standard, was performed selectively (4%) but demonstrated a high positivity rate (64%), consistent with its role as a confirmatory test reserved for patients with strong clinical suspicion. These findings underscore a persistent diagnostic gap in which routine modalities cannot reliably exclude clinically significant cardiac injuries.

Within this diagnostic gap, troponin T offers an important adjunct, as a rapid test accessible even after hours, which addresses a practical need in LMIC emergency departments. To date, only one retrospective study at CMJAH has evaluated troponins in occult cardiac injuries, reporting a sensitivity of 19.7%, specificity of 98.2%, PPV of 85.7% and NPV of 69.2%.^5^ In comparison, our study demonstrated substantially higher sensitivity (90.5% pooled against definitive modalities) but lower specificity (37.5% pooled), with a comparable NPV and lower PPV. Differences in case mix, timing of troponin sampling and injury severity likely account for this variation. The high sensitivity observed in our cohort supports the value of troponin T as a screening test; however, its moderate NPV against definitive modalities indicates that a negative result alone cannot reliably exclude cardiac injury. Conversely, the low specificity reflects the non-specific elevation of troponins in the context of associated thoracic trauma. These findings reinforce the role of troponin T as an adjunct within a multimodal diagnostic approach, particularly in settings where the subxiphoid pericardial window is not readily accessible.^5,19^

Although lactate and base excess are established markers of hypoperfusion and metabolic acidosis, these derangements were less pronounced in our stable population. This contrasts with studies involving unstable or mixed trauma populations, where metabolic markers showed stronger predictive value.^16,25^ In our cohort, troponin and lactate trended towards reflecting greater injury severity, but their predictive utility appeared reduced in haemodynamically stable patients. Baseline troponin was higher in non-survivors; however, this difference was not statistically significant, likely reflecting the low mortality rate (3.9%). Mortality was instead strongly linked to structural findings, haemopericardium and pleural injury, demonstrating that in stable patients, anatomical evidence of injury is a stronger predictor of poor outcomes than biochemical markers alone.

### Strengths and limitations

The principal strength of this study is the size of the cohort, which represents the largest series of haemodynamically stable patients with penetrating chest trauma evaluated for troponin T reported to date. The study uniquely addresses a key literature gap, as most existing evidence focuses on blunt chest trauma or haemodynamically unstable penetrating injuries. The real-world setting captures the patterns of a high-burden trauma environment, and a comprehensive range of variables permitted thorough assessment of diagnostic accuracy and predictors of morbidity and mortality.

Several limitations must be acknowledged. The retrospective design introduced potential selection bias, as patients presenting to the hospital may not represent all PTCI victims. Reliance on existing records affected data completeness; in particular, serial troponin measurements were not uniformly available. Information bias may have arisen from changes in documentation practices and a lack of homogeneity among clinical staff over the study period. Pre-existing medical conditions that may have confounded troponin levels were not systematically recorded. The majority of patients were lost to follow-up, precluding assessment of late complications such as pericarditis or arrhythmias. Finally, as a single-centre study, generalisability to other South African and international settings may be limited.

An important methodological limitation must be acknowledged in relation to Table 5. Per standards for reporting diagnostic accuracy studies (STARD) 2015 guidelines, diagnostic accuracy should be evaluated against a validated reference standard. Table 3 and Table 4 present true diagnostic accuracy (troponin T versus subxiphoid pericardial window and intraoperative findings, respectively). In contrast, Table 5 compares troponin T with other non-reference screening modalities (eFAST, ECG, lactate, base excess), yielding inter-test concordance rather than true diagnostic accuracy. The NPV figures from Table 5 may be artificially elevated if the comparator modalities themselves have low sensitivity. We have therefore reframed Table 5 as a concordance analysis and anchored all formal diagnostic accuracy claims to Table 3 and Table 4.

### Implications and recommendations

These findings suggest that troponin T may serve as a useful screening adjunct in the initial assessment of haemodynamically stable patients with PTCI, particularly where advanced imaging and surgical expertise are limited. However, the low specificity (37.5% pooled) and moderate NPV (60.0%) against definitive modalities indicate that troponin T should not be used as a standalone diagnostic tool. A negative troponin result, when combined with negative eFAST and a normal ECG, may identify a lower-risk subset warranting close observation rather than immediate surgical exploration; conversely, a positive troponin warrants further investigation regardless of other modality results. Implementation would require validation of cost-effectiveness in resource-limited settings, as false-positive results may drive unnecessary surgical consultation or imaging. Future prospective multicentre studies should: (1) establish optimal troponin T cut-off values and timing of serial measurements specific to penetrating trauma; (2) evaluate receiver-operating characteristic curves to optimise sensitivity–specificity trade-offs; (3) assess the clinical impact of troponin-guided triage algorithms on patient outcomes, length of stay, and resource utilisation; and (4) validate findings across diverse resource settings in South Africa and other LMICs.

## Conclusion

In this large single-centre cohort, troponin T demonstrated high sensitivity (90.5% pooled against reference standards) for detecting occult cardiac injury in haemodynamically stable patients with penetrating chest trauma. Although its low specificity limits its use as a standalone diagnostic tool, troponin T can support early risk stratification and guide the judicious use of further diagnostic and surgical resources. These findings are particularly relevant to South African emergency departments and other resource-constrained settings where rapid, inexpensive screening tools are critically needed.
